# Extracts of *Artocarpus communis* Induce Mitochondria-Associated Apoptosis via Pro-oxidative Activity in Human Glioblastoma Cells

**DOI:** 10.3389/fphar.2018.00411

**Published:** 2018-05-02

**Authors:** Chiang-Wen Lee, Lee-Fen Hsu, Ming-Hsueh Lee, I.-Ta Lee, Ju-Fang Liu, Yao-Chang Chiang, Ming-Horng Tsai

**Affiliations:** ^1^Division of Basic Medical Sciences, Department of Nursing, Chang Gung University of Science and Technology, Chiayi, Taiwan; ^2^Chronic Diseases and Health Promotion Research Center, Chang Gung University of Science and Technology, Chiayi, Taiwan; ^3^Research Center for Industry of Human Ecology and Research Center for Chinese Herbal Medicine, College of Human Ecology, Chang Gung University of Science and Technology, Taoyuan, Taiwan; ^4^Department of Respiratory Care, Chang Gung University of Science and Technology, Chiayi, Taiwan; ^5^Division of Neurosurgery, Department of Surgery, Chang Gung Memorial Hospital, Chiayi, Taiwan; ^6^The Center of Translational Medicine, Department of Education and Research, Taichung Veterans General Hospital, Taichung, Taiwan; ^7^Central Laboratory, Shin Kong Wu Ho-Su Memorial Hospital, Taipei, Taiwan; ^8^Center for Drug Abuse and Addiction, China Medical University Hospital, China Medical University, Taichung, Taiwan; ^9^Department of Pediatrics, Division of Neonatology and Pediatric Hematology/Oncology, Yunlin Chang Gung Memorial Hospital, Yunlin County, Taiwan; ^10^College of Medicine, Chang Gung University, Taoyuan, Taiwan

**Keywords:** apoptosis, artocarpin, caspase, glioblastoma multiforme, ROS

## Abstract

Glioblastoma multiforme (GBM) is an extremely aggressive and devastating malignant tumor in the central nervous system. Its incidence is increasing and the prognosis is poor. Artocarpin is a natural prenylated flavonoid with various anti-inflammatory and anti-tumor properties. Studies have shown that artocarpin is associated with cell death of primary glioblastoma cells. However, the *in vivo* effects and the cellular and molecular mechanisms modulating the anticancer activities of artocarpin remain unknown. In this study, we demonstrated that treating the glioblastoma cell lines U87 and U118 cells with artocarpin induced apoptosis. Artocarpin-induced apoptosis is associated with caspase activation and poly (ADP-ribose) polymerase (PARP) cleavage and is mediated by the mitochondrial pathway. This is associated with mitochondrial depolarization, mitochondrial-derived reactive oxidative species (ROS) production, cytochrome c release, Bad and Bax upregulations, and Bcl-2 downregulation. Artocarpin induced NADPH oxidase/ROS generation plays an important role in the mitochondrial pathway activation. Furthermore, we found artocarpin-induced ROS production in mitochondria is associated with Akt- and ERK1/2 activation. After treatment with artocarpin, ROS causes PI3K/Akt/ERK1/2-induced cell death of these tumor cells. These observations were further verified by the results from the implantation of both U87 and U118 cells into *in vivo* mouse. In conclusion, our findings suggest that artocarpin induces mitochondria-associated apoptosis of glioma cells, suggesting that artocarpine can be a potential chemotherapeutic agent for future GBM treatment.

## Introduction

Malignant glioma is the most common and lethal primary tumor of the central nervous system. Among them, GBM accounts for more than half of these cases with a short median survival of only 15 months (Alifieris and Trafalis, 2015). Despite substantial advances in cancer treatment, mainly surgical resection followed by chemotherapy and adjuvant radiation therapy (Juratli et al., 2013; Carlsson et al., 2014), GBM remains the most deadly form of brain tumor because of its location, the devastating nature, influence of the blood-brain barrier and therapeutic resistance (Alifieris and Trafalis, 2015). Various new treatment strategies, including immunotherapy, nanotechnology, and molecular-targeted therapy have emerged as potential therapeutic options. However, these treatment strategies still possess limited success and do not have a substantial clinical effect (Polivka et al., 2012; Bambury and Morris, 2014; Ung and Yang, 2015).

Reactive oxygen species (ROS) is well known to play an important role in various pathophysiological pathways including cell death, apoptosis, and cell proliferation (Montezano and Touyz, 2014). Although excessive ROS will cause cell damage or death, the oxidative stress caused by ROS can be utilized as a novel cancer-damaging adjuvant (Bauer, 2012; Vallejo et al., 2017). Several recent studies reported that targeting ROS and driving highly selective apoptotic signaling pathways have been proven effective against certain cancers (Zou et al., 2015; Chen W. et al., 2016). Various biochemical pathways are involved in ROS-mediated cancer cell apoptosis, and several studies have focused on plant-derived compounds in recent years (Seebaluck et al., 2015; Li et al., 2016; Vallejo et al., 2017). Since GBM is difficult to treat, utilizing natural products with chemotherapeutic effects have been applied in the control of high-grade gliomas (Perry M.C. et al., 2010; Yin et al., 2014). However, a recent study indicated that these treatments provided limited survival benefit (Perry J.R. et al., 2010). Therefore, increasing efforts are being made to investigate tumor pathophysiology and the molecular mechanisms in order to optimize therapeutic outcomes.

Artocarpin is a bioactive plant component derived from *Artocarpus communis*. This plant is widely distributed in tropical countries. Artocarpin exhibits a broad spectrum of pharmacological properties including synergistic antibacterial- and antioxidant activities, and anticancer and anti-inflammatory effects (Perry J.R. et al., 2010; Septama and Panichayupakaranant, 2016). Artocarpin may exert anticancer activity by certain apoptosis pathways. It interrupts cell proliferation, invasion, and angiogenesis (Arung et al., 2010a,b; Hu et al., 2015). Furthermore, artocarpin has also been reported to induce programmed cancer cell death by modulating the MAPK- and Akt/mTOR pathways (Hu et al., 2015). Nevertheless, the molecular mechanisms underlying the pharmacological properties and therapeutic effects of artocarpin on GBM remain unknown.

In the present study, we aimed to investigate the anticancer mechanisms of artocarpin on GBM. The pro-apoptotic effects of artocarpin in human glioblastoma cells, U87 and U118, are mediated by increased ROS production, activation of the Akt/ERK1/2 phosphorylation cascade, and the mitochondrial pathway. These observations were corroborated by the implantation of U87 and U118 cells in mice. To the best of our knowledge, the present study is the first to demonstrate the potential of artocarpin as a chemotherapeutic agent for the treatment of GBM.

## Materials and Methods

### Materials

MCI-186, U0126, PD98059, LY294002, SH-5, NAC, and DPI were purchased from Biomol Research Laboratories Inc. (Plymouth Meeting, PA, United States). Z-VAD-FMK and MitoTEMPOL were purchased from Sigma-Aldrich Corp. (St. Louis, MO, United States). Anti-GAPDH (#SC-32233), anti-Akt (#SC-8312), anti-phospho-Akt (#SC-7985) and anti-COX IV antibodies and APO were purchased from Santa Cruz Biotechnology, Inc. (Dallas, TX, United States). Anti-caspase-3 (#9662S), anti-caspase-7 (#12827S), anti-caspase-9 (#9508S), anti-Bax (#9239S), anti-Bad (#5023S), anti-Bcl-2 (#15071S) and anti-phospho-ERK1/2 (#SC-8312) antibodies were purchased from Cell Signaling Technology Inc. (Denver, MA, United States). Anti-PARP (#13371-AP) was purchased from Proteintech Group (Manchester, United Kingdom), and anti-cytochrome C (#ab133504) was purchased from Abcam (Cambridge, United Kingdom). Artocarpin was purchased from Pulin Biotech Company Limited (Taipei, Taiwan). Artocarpin purity was determined by high-performance liquid chromatography (HPLC) and found to be >98%.

### Mice Tumor Xenograft Study

Six-week-old male mice with severe combined immunodeficiency [BALB/cA-nu (nu/nu)] were obtained from the National Science Council and Animal Center (Taichung, Republic of China) and these mice were kept in a pathogen free environment. U87 and U118 cells (1 × 10^6^ cells 200 μL normal saline) were injected subcutaneously into the flanks of mice. Tumors were allowed to grow for 14 days. By that time, the tumors were visible on the outer body surface. Ten mice per group were treated with either vehicle or 2 mg/kg artocarpin daily for 21 days. We determined the tumor volume twice a week using a caliper, according to the formula *V* = (LW2) p/6: where *V* = volume (mm^3^), *L* = biggest diameter (mm), *W* = smallest diameter (mm). All animal studies were conducted in accordance with institutional guidelines and the protocol was approved by the Animal Care Committee of Shin Kong Wu Ho-Su Memorial Hospital in Taipei, Taiwan.

### Cell Culture

U87 and U118 human glioblastoma cells were purchased from the American Type Culture Collection (ATCC, Manassas, VA, United States). The U87 and U118 cells were cultured in Dulbecco’s Modified Eagle Medium/Nutrient Mixture F-12(DMEM/F-12) (Life Technologies Group, Grand Island, NY, United States) supplemented with 10% fetal bovine serum (FBS) (Hazelton Research Products, Reston, VA, United States) and 1% penicillin–streptomycin at 37°C in 5% CO_2_. The medium was replenished every 2 days and the cells were subcultured every 4 days.

### Cell Viability

We measured cell viability according to the formation of formazan; a blue product resulted from the metabolism of a colorless substrate by mitochondrial dehydrogenases. U87 and U118 cells, rat brain cortex astrocytes, or mouse microglial cells (2.5 × 10^5^ per well in a 24-well plate) were incubated at 37°C with various concentrations of artocarpin. These cells were then treated with a 5 mg/mL solution of MTT [3-(4,5-dimethylthiazol-2-yl)-2,5-diphenyltetrazolium bromide] purchased from Sigma-Aldrich Corp. (St. Louis, MO, United States) for 2 h. A microplate reader was used to measure the dark blue formazan crystals formed in intact cells dissolved in dimethylsulfoxide (DMSO; Sigma-Aldrich Corp., St. Louis, MO, United States). The absorbance of the resultant solution was measured at λ = 540 nm. The results were expressed as percentages of MTT metabolized in the artocarpin-treated cells relative to those of the control cells.

### Preparation of Cell Extracts and Western Blot

The U87 and U118 cells were grown to confluence in a six-well plate, and then treated with artocarpin (10 μM) at various time intervals. The cells were then collected and placed in ice-cold lysis buffer containing 25 mM Tris-HCl (pH 7.4), 25 mM NaCl, 25 mMNaF, 25 mM sodium pyrophosphate, 1 mM sodium vanadate, 2.5 mM EDTA, 0.05% (w/v) Triton X-100, 0.5% (w/v) sodium dodecyl sulfate (SDS), 0.5% (w/v) deoxycholate, 0.5% (w/v) NP-40, 5 μ g/ml leupeptin, 5 μ g/ml aprotinin, and 1 mM phenylmethylsulfonyl fluoride (PMF). Lysates were centrifuged at 45,000 × *g* for 1 h at 4°C and whole cell extracts were obtained according to methods described in previous studies (Lee et al., 2014). Samples were denatured, subjected to SDS-PAGE on a 12% running gel, and transferred to a nitrocellulose membrane. The membranes were incubated with anti-caspase-3, anti-caspase-7, anti-caspase-9, anti-PARP, anti-Bcl-2, anti-Bax, or anti-Bad antibody for 24 h. They were then incubated with anti-mouse or anti-rabbit horseradish peroxidase antibody for 1 h. Enhanced chemiluminescent (ECL) reagents purchased from PerkinElmer Inc. (Waltham, MA, United States) were used to detect immunoreactive bands These were developed with Hyperfilm-ECL from PerkinElmer Inc. (Waltham, MA, United States).

### Caspase Activity Determinations

Caspase-3, -7, and -9 colorimetric assay kits (R&D Systems Inc., Minneapolis, MN, United States) were used to measure the caspase activity in the cell lysates. The cells were treated with artocarpin for 24 h, and then lysed in a buffer mixture [50 mM Tris-HCl (pH 7.4), 2 mM DTT, 1 mM EDTA, 10 mM digitonin, and 10 mM EGTA]. Ac-DEVD-pNA and Ac-LEHD-pNA were used as casepase-3, -7, and -9 substrates for the incubation of the cell lysate at 37°C for 1 h. Caspase activity and absorbance were measured using an enzyme-linked immunosorbent assay (ELISA) reader at OD_405_. Three independent experiments were run for these analyses.

### Cytosolic and Mitochondrial Protein Extraction

All cells were treated with a digitonin buffer (20 mM Hepes-KOH, 110 mM KAc, 2 mM MgAc2, 5 mM NaAc, 1 mM EGTA, and 200 μg/ml digitonin at pH 7.3) on ice for 10 min to obtain cytosolic and mitochondrial fractions. The cell lysate was centrifuged at 10,000 × *g* at 4°C for 15 min. The supernatant was collected as the cytosolic fraction. The pellet, which contained the mitochondrial fraction, was resuspended in 1X-SDS loading buffer. Protein content was estimated with a commercial protein assay (Bio-Rad Laboratories, Hercules, CA, United States). The samples were either stored at -80°C or analyzed immediately. Western blot was run to analyze the total, cytosolic, and mitochondrial extracts.

### Flow Cytometry Apoptosis Analysis

Flow cytometric assessments of cell viability and apoptosis were determined with a FITC-annexin V/propidium iodide assay (Thermo Fisher Scientific, Waltham, MA, United States). Briefly, growing U87 and U118 cells were digested with 0.25% trypsin and counted. These cells were then diluted to a final concentration of 1 × 10^5^ cells/ml and inoculated in a culture dish at a density of 10 mL/dish. The cells were then treated with artocarpin for the indicated times. Cells were detached by incubation with trypsin-EDTA and centrifuged at 1,500 rpm for 5 min. The pellet was collected and resuspended in 100 μL annexin-binding buffer. FITC-annexin V and propidium iodide were then added to the cell suspension. After incubation at room temperature for 15 mins, the stained cells were analyzed by flow cytometry (FACScan; Becton Dickinson, Franklin Lakes, NJ, United States). Fluorescence emission was measured at λ = 530 nm (excitation) and at λ = 575 nm (emission).

### Mitochondrial Membrane Potential Detection

Mitochondrial membrane potential (ΔΨm) was measured using the fluorescent dye JC-1 (5,5′,6,6′-tetrachloro-1,1′,3,3′-tetraethylbenzimidazolyl-carbocyanine iodide) (Sigma-Aldrich Corp., St. Louis, MO, United States). The change from red- to green fluorescence in the JC-1 assay was used to evaluate the decline in mitochondrial membrane potential. This parameter was used as an early indicator of apoptosis. Various concentrations of artocarpin were used to treat U87 and U118 cells for 24 h. They were then washed twice with PBS. One milliliter serum-free DMEM/F-12 medium and 1 mL JC-1 staining working solution were added to each sample. The plate was incubated for 20 min at 37°C in 5% CO_2_ and the cells were observed and photographed under a fluorescence microscope (Axiovert 200M, Carl Zeiss AG, Oberkochen, Germany). The JC-1 monomers were detected at λ = 514 nm (excitation) and λ = 529 nm (emission). JC-1 aggregates were detected at λ = 585 nm (excitation) and λ = 590 nm (emission). Change in mitochondrial membrane potential (ΔΨm) was represented by the ratio of red- to green fluorescence. These assays were repeated five times.

### Determination of NADPH Oxidase Activity by Chemiluminescence Assay

After incubation, the cells were gently scraped and centrifuged them at 400 × *g* and 4°C for 10 min. The cell pellet was resuspended in 35 μl/per well of ice-cold RPMI-1640 medium. The cell suspension was stored on ice. Either NADPH (1 μM) (Sigma-Aldrich Corp., St. Louis, MO, United States) or lucigenin (20 μM) (Sigma-Aldrich Corp., St. Louis, MO, United States) was added to a 5 μl of cell suspension (0.2 × 10^5^ cells) to initiate the reaction. The final RPMI-1640 medium volume was 200 μl. Chemiluminescence was immediately measured in an Appliskan luminometer (Thermo Fisher Scientific, Waltham, MA, United States) using an out-of-coincidence mode. The appropriate blanks and controls were established. Both NADPH and NADH contributed to the background of the lucigenin chemiluminescence (James et al., 2005; Manning and Cantley, 2007; Sahu et al., 2009; Hsieh et al., 2010; Kim et al., 2013; Savitskaya and Onishchenko, 2015; Chen Z.P. et al., 2016; Lee et al., 2017; Tsai et al., 2017; Xie et al., 2017) counts per min). Chemiluminescence was measured continuously for 12 min and expressed in the standard units of NADPH oxidase activity.

### Measurement of Mitochondrial-Derived ROS Production

MitoSOX Red mitochondrial superoxide indicator was purchased from Thermo Fisher Scientific (Waltham, MA, United States) and used to detect fluorescence from mitochondrial-derived ROS production. U87 and U118 cells were washed with warm Hanks’ Balanced Salt Solution (HBSS) and incubated at 37°C for 30 min in a cell medium containing 5 μM MitoSOX Red mitochondrial superoxide indicator. The HBSS or the MitoSOX Red mitochondrial superoxide indicator medium was removed and replaced with fresh medium. The U87 and U118 cells were incubated with artocarpin for various time periods. The cells were then washed twice with PBS and detached with EDTA/trypsin. They were then run through a FACScan flow cytometer (Becton Dickinson, Franklin Lakes, NJ, United States) and the fluorescence was measured at λ = 510 nm (excitation) and λ = 580 nm (emission).

### Measurement of Intracellular ROS Accumulation

CellROX Green Reagent (Thermo Fisher Scientific, Waltham, MA, United States), a novel fluorogenic probe of cellular oxidative stress, was used in this assay. CellROX Green Reagent fluorescence was measured at λ = 485 (excitation) and at λ = 520 nm (emission). The cells were observed under a fluorescence microscope (Axiovert 200M; Carl-Zeiss AG, Oberkochen, Germany). U87 or U118 cells were washed with warm PBS and incubated in HBSS containing 5 μM CellROX Green Reagent at 30°C for 30 min. The HBSS or CellROX Green Reagent medium was removed and replaced with fresh medium. U87 and U118 cells were incubated with artocarpin for various time periods. The cells were then washed twice with PBS, detached with trypsin/EDTA, and run through a FACScan flow cytometer (Becton Dickinson, Franklin Lakes, NJ, United States) to determine fluorescence at λ = 485 nm (excitation) and λ = 520 nm (emission).

### Data Analysis

All results were processed with GraphPad Prism Program (GraphPad, San Diego, CA, United States). Quantitative data are expressed as the means ± SE. One-way ANOVA and Tukey’s *post hoc* test were used to identify significant differences among treatments. A *P-*value of less than 0.05 was considered statistically significant.

## Results

### Artocarpin Decreases Viability and Induces Apoptosis in U87- and U118 Cells by Activating Caspase and Cleaving PARP

**FIGURE 1:**
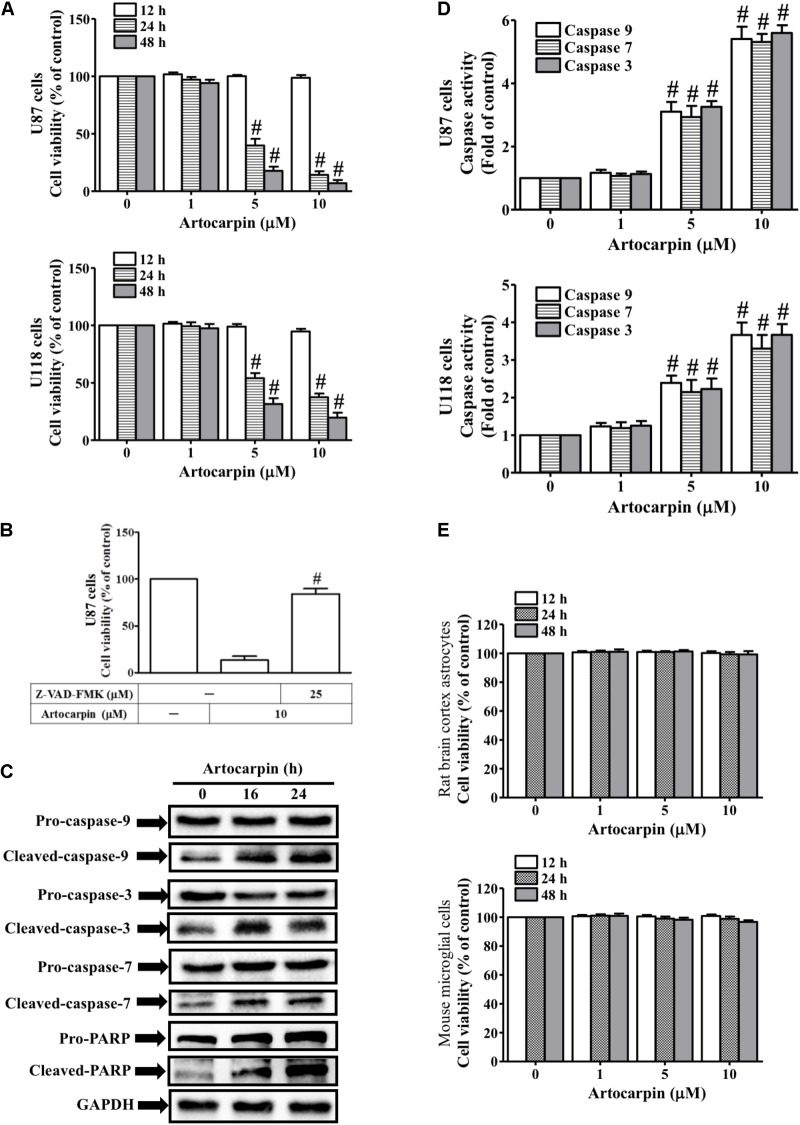
Artocarpin decreases cell viability and induces apoptosis in U87 or U118 cells by caspase activation and PARP cleavage. **(A)** Cells were treated with various concentrations of artocarpin for the indicated time intervals. Cell viability was assayed with MTT. **(B)** Cells were pretreated with Z-VAD-FMK (25 μM) for 1 h then incubated with artocarpin for 24 h. Cell viability was assayed with MTT. **(C)** Cells were treated with artocarpin (10 μM) for 16- or 24 h. Cleaved caspase-3, -7, -9 and PARP protein expression levels were determined by western blot. **(D)** Cells were treated with various artocarpin concentrations for 24 h. Caspase activity was analyzed with caspase-3, -7, and -9 colorimetric assay kits. **(E)** Rat brain cortex astrocytes and mouse microglial cells were treated with various artocarpin concentrations for the indicated times. Cell viability was assayed with MTT. Data are expressed as means ± SE of three independent experiments. ^#^*P* < 0.01 compared with cells exposed to vehicle **(A,D)**. ^#^*P* < 0.01 compared with cells exposed to artocarpin alone **(B)**.

We investigated the antitumor potential of artocarpin by evaluating its effects on U87- and U118 cell viability (**Figure 1A**). We used MTT assay to measure viability after treating the cells with various artocarpin concentrations (1–10 μM) over different time periods. Artocarpin significantly decreased U87- and U118 cell viability in a dose- and time-dependent manner. Relative to the control, significant reductions in viability were noted at 5 μM. Viability decreased by ≤70% relative to the control with 10 μM artocarpin (**Figure 1A**). Caspase activity and apoptosis were assessed in artocarpin-treated U87- and U118 cells. Their viabilities were determined after treatment with the pan-caspase inhibitor Z-VAD-FMK. **Figure 1B** shows that 10 μM artocarpin significantly decreased the viability of the U87 cells relative to the control. Z-VAD-FMK (25 μM) abrogated the effect of artocarpin. Therefore, apoptosis is associated with caspase activation. Western blot was run to determine the expression levels of caspase-3, -7, -9, and PARP cleavage after exposures to artocarpin at various time periods (**Figure 1C**). The assay confirmed that these effector caspases were involved in this process. However, pro-caspase-3, -7, and -9 and PARP did not significantly change relative to the control after artocarpin treatment. We also investigated the effects of artocarpin on caspase-3, -7, and -9 activities in U87- and U118 cells. **Figure 1D** shows that both 5 μM and 10 μM artocarpin significantly increased caspase-3, -7, and -9 activities. It induced these three enzymes after 16 h, so apoptosis was mediated by the mitochondrial pathway. We then evaluated the effects of artocarpin on normal rat brain cortex astrocyte- and mouse microglial cell viability. Artocarpin did not significantly affect the viability of these cells (**Figure 1E**). We also used anti-TRAIL-PE antibody and flow cytometry to identify the extrinsic apoptosis pathway. As shown in Supplementary Figure S5, the treatment of U87 cells with 10 mM artocarpin for 1–24 h had no significant effect on death receptor expression. Therefore, these data suggest that artocarpin activates caspases and cleaves PARP to decrease cell viability and induce apoptosis in U87- and U118 cells.

### Artocarpin-Induced Apoptosis in U87 or U118 Cells Is Mediated by ROS Generation

**FIGURE 2:**
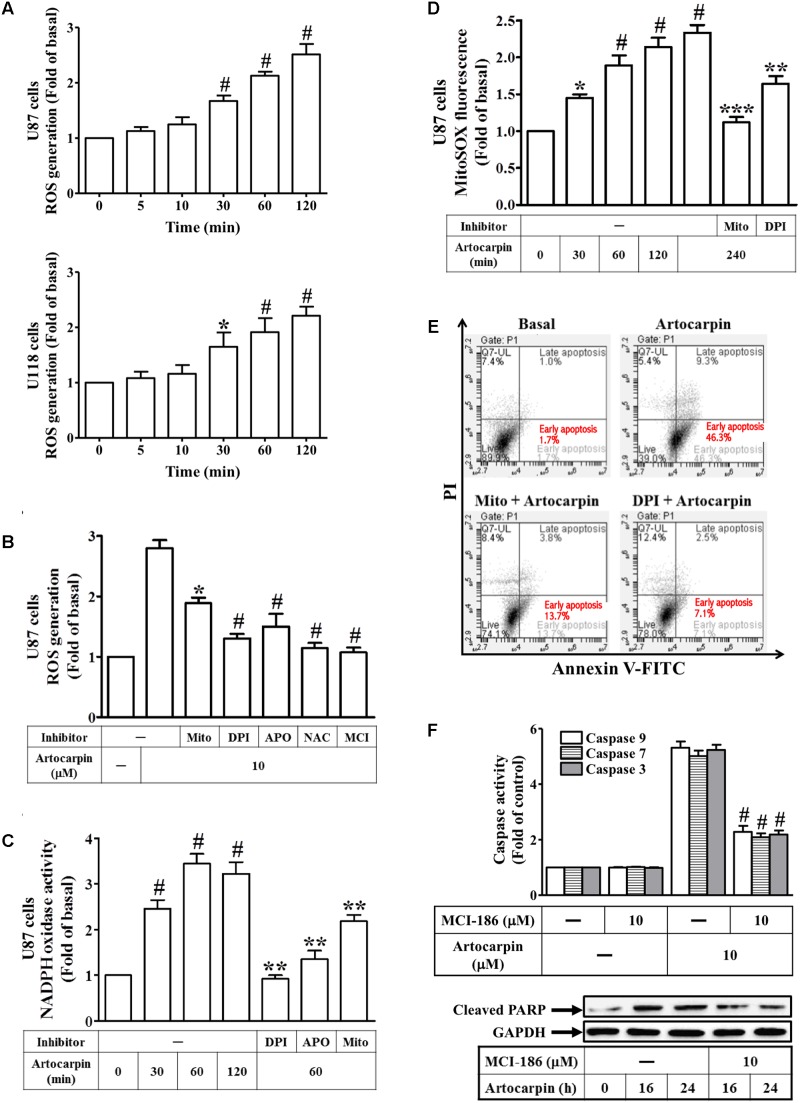
Artocarpin-induced apoptosis in U87- or U118 cells is mediated by ROS generation. **(A)** Cells were treated with artocarpin (10 μM) for the indicated times. ROS generation was measured with CellROX reagent. **(B)** Cells were pretreated with MitoTEMPOL (10 μM), DPI (1 μM), APO (100 μM), NAC (5 mM), or MCI-186 (10 μM) for 1 h then incubated with artocarpin for 2 h. ROS generation was measured with CellROX reagent. **(C)** Cells were either not pretreated or pretreated with DPI (1 μM), APO (100 μM), or MitoTEMPOL (10 μM) for 1 h then incubated with artocarpin for the indicated times. NADPH oxidase activity was measured. **(D)** Cells were either not pretreated or pretreated with MitoTEMPOL (10 μM) or DPI (1 μM) for 1 h then incubated with artocarpin for the indicated times. MitoSOX fluorescence was measured with a fluorescence plate reader. **(E)** Cells were pretreated with MitoTEMPOL (10 μM) or DPI (1 μM) for 1 h then incubated with artocarpin for 24 h. U87 cell apoptosis was evaluated by flow cytometry. U87 cells were labeled with annexin V-FITC and PI. Flow cytometry profile shows annexin-V-FITC staining in the *x*-axis and PI in the *y*-axis. **(F)** Cells were pretreated with MCI-186 (10 μM) for 1 h then incubated with artocarpin for 24 h. Caspase activity was determined with caspase-3, -7, and -9 colorimetric assay kits. Cells were also pretreated with MCI-186 (10 μM) for 1 h then incubated with artocarpin for the indicated times. Cleaved PARP protein expression was determined by western blot. Data are expressed as means ± SE of three independent experiments. ^#^*P* < 0.01 compared with cells exposed to vehicle **(A,C,D)**. ^∗^*P* < 0.05, ^#^*P* < 0.01 compared with cells exposed to artocarpin alone **(B,F)**. ^∗∗^*P* < 0.01 compared with cells exposed to artocarpin alone **(C,D)**. ^∗∗∗^*P* < 0.001.

We investigated the effect of artocarpin on intracellular ROS production in U87- and U118 cells. U87- and U118 cells were treated with artocarpin at various intervals between 5 and 120 min. OS production was measured by flow cytometry of the CellROX Green Reagent fluorescence signal (**Figure 2A**). We found that intracellular ROS levels increased as soon as 30 min after treatment. These ROS may have been produced by NADPH oxidases, xanthine oxidase, cyclooxygenase, lipoxygenase, mitochondrial electron transport enzymes, or uncoupled nitric oxide synthase (Brentnall et al., 2013). In the present study, we showed that artocarpin-induced ROS production was effectively attenuated by pretreating U87 cells with MitoTEMPOL (a water-soluble mitochondrion-targeted antioxidant), DPI (NADPH oxidase inhibitor), APO (a specific inhibitor of NADPH oxidase), NAC (a thiol-containing antioxidant), or MCI-186 (a free radical scavenger) (**Figure 2B**). As shown in Supplementary Figure S4B, treatment with inhibitor alone has no significant effects on each experiment. We also demonstrated that artocarpin enhanced NADPH oxidase activity in a time-dependent manner but this effect was reduced by pretreatment with DPI, APO, or MitoTEMPOL (**Figure 2C**). We also investigated whether artocarpin induced mitochondrial superoxide in U87 cells. As shown in **Figure 2D**, artocarpin significantly induced mitochondrial ROS in a time-dependent manner. This effect was inhibited by preincubation with MitoTEMPOL or DPI. On the other hand, U0126, LY294002, and zVAD pretreatments did not suppress artocarpin-induced increases in mitochondrial superoxide in U87 cells (Supplementary Figure S1). We also explored whether an increase in ROS production was critical for artocarpin-induced apoptosis. Cells either received no pretreatment or they were pretreated with MitoTEMPOL or DPI. They were then exposed to artocarpin for 24 h and analyzed for apoptosis. **Figure 2E** shows that artocarpin-induced apoptosis was significantly reduced relative to the control in the presence of either MitoTEMPOL or DPI. We also examined the role of ROS in artocarpin-induced caspase activation and PARP cleavage. As shown in **Figure 2F**, artocarpin-induced caspase-3, -7, and -9 activation and PARP cleavage were reduced in both U87 and U118 cells relative to the control by pretreatment with MCI-186. As shown in Supplementary Figure S4A, treatment with inhibitor alone has no significant effects on each experiment. These results, then, indicate that artocarpin induces apoptosis in U87 and U118 by activating intracellular ROS.

### Artocarpin Induces Apoptosis via the Mitochondrial Pathway in U87 and U118 Cells

**FIGURE 3:**
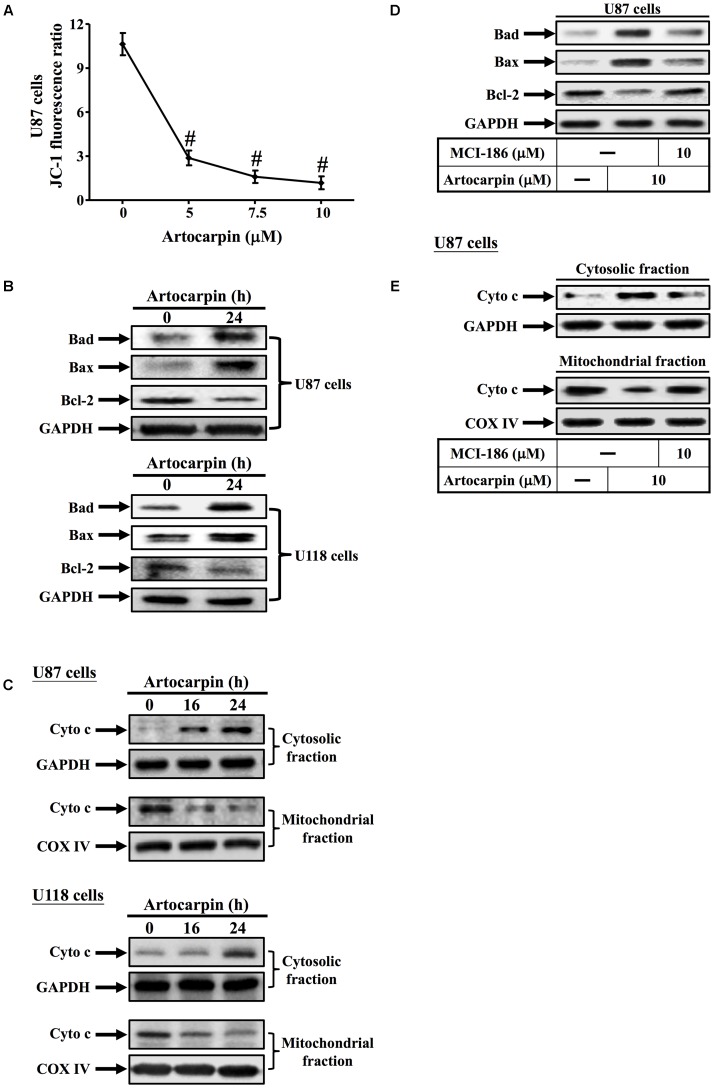
Artocarpin induces apoptosis via the mitochondrial pathway. **(A)** Cells were treated with various artocarpin concentrations for 24 h. Effect of artocarpin on U87 mitochondrial depolarization was measured with JC-1 staining and a fluorescence plate reader. **(B)** Cells were treated with artocarpin for 24 h then the Bad, Bax, and Bcl-2 protein expression levels were determined by western blot. **(C)** Cells were treated with artocarpin for 16- or 24 h. Cytosolic- and mitochondrial fractions were prepared and subjected to western blot with anti-cytochrome c antibody. GAPDH was used as a marker protein for cytosolic fractions. COX IV was used as a marker protein for mitochondrial fractions. **(D)** Cells were pretreated with MCI-186 (10 μM) for 1 h then treated with artocarpin for 24 h. Bad, Bax, and Bcl-2 protein expression levels were determined by western blot. **(E)** Cells were pretreated with MCI-186 (10 μM) for 1 h then treated with artocarpin for 24 h. Cytosolic- and mitochondrial fractions were prepared and subjected to western blot with anti-cytochrome c antibody. Data are expressed as means ± SE of three independent experiments. ^#^*P* < 0.01 compared with cells exposed to vehicle.

In this study, we used the fluorescent dye JC-1 to measure the mitochondrial membrane potential (ΔΨm) of artocarpin-treated U87 cells. Changes in mitochondrial membrane potential (ΔΨm) were represented by the ratio of red–green fluorescence. **Figure 3A** shows that the JC-1 fluorescence ratio significantly decreased after 24 h in a dose-dependent manner. The mitochondrion-mediated apoptosis pathway is regulated by the Bcl-2 family of antiapoptotic (Bcl-2, Bcl-XL, and Bcl-1) and proapoptotic (Bax, Bad, and Bak) proteins (Seebaluck et al., 2015). We demonstrated that artocarpin induced Bad- and Bax expression and repressed Bcl-2 expression in both U87- and U118 cells (**Figure 3B**). Western blot analysis revealed that the transfer of cytochrome c from the mitochondria to the cytosol in U87- and U118 cells following 16- or 24 h artocarpin treatment altered mitochondrial membrane permeability (**Figure 3C**). Artocarpin-induced expression of cytochrome c was not inhibited by pretreatment with LY294002 or U0126 (Supplementary Figure S2A). We also showed that MCI-186 pretreatment significantly downregulated artocarpin-induced Bad- and Bax and upregulated artocarpin-repressed Bcl-2 in U87 cells (**Figure 3D**). On the other hand, MCI-186 pretreatment also repressed the artocarpin-induced movement of cytochrome c from the mitochondria to the cytosol (**Figure 3E**). These data, then, suggest that artocarpin induces apoptosis in U87- and U118 cells via the mitochondrial pathway.

### ROS Induces Akt Activation Leading to Apoptosis of U87 Cells

**FIGURE 4:**
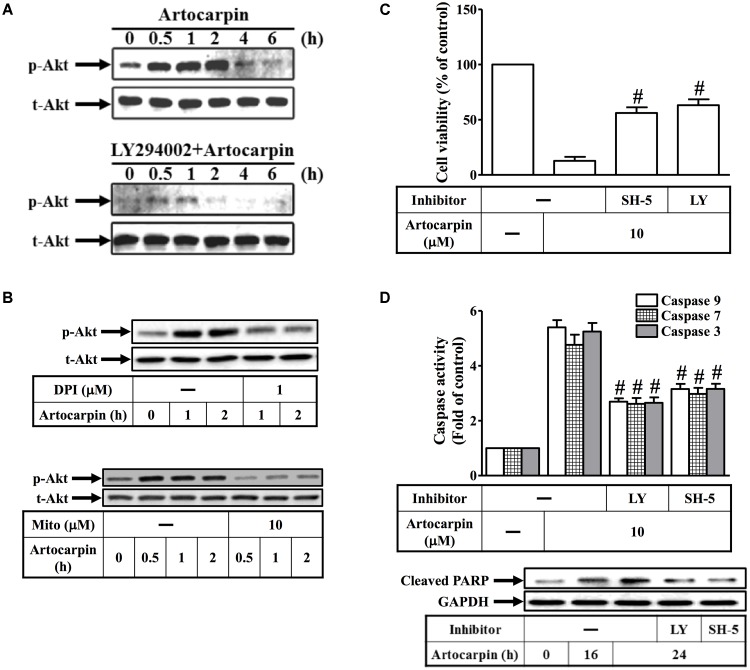
Reactive oxidative species induces Akt activation resulting in the apoptosis of U87 cells. **(A)** Cells were either not pretreated or pretreated with LY294002 (10 μM) for 1 h then incubated with artocarpin for the indicated times. Phospho-Akt protein expression was determined by western blot. **(B)** Cells were either not pretreated or pretreated with DPI (1 μM) or MitoTEMPOL (10 μM) for 1 h then incubated with artocarpin for the indicated times. Phospho-Akt protein expression was determined by western blot. **(C)** Cells were pretreated with SH-5 (1 μM) or LY294002 (10 μM) for 1 h then incubated with artocarpin for 24 h. Cell viability was assayed with MTT. **(D)** Cells were pretreated with LY294002 (10 μM) or SH-5 (1 μM) for 1 h then incubated with artocarpin for 24 h. Caspase activity was analyzed with caspase-3, -7, and -9 colorimetric assay kits. Cells were pretreated with LY294002 (10 μM) or SH-5 (1 μM) for 1 h then incubated with artocarpin for the indicated times. Cleaved PARP protein expression was determined by western blot. Data are expressed as means ± SE of three independent experiments. ^#^*P* < 0.01 compared with cells exposed to artocarpin alone.

There is growing evidence that certain flavonoids induce apoptosis by modifying various signal transduction pathways like PI3K/Akt and MAPKs (Hu et al., 2015). We observed that artocarpin activated Akt but this activation was repressed by pretreatment with the PI3K inhibitor LY294002 (**Figure 4A**). However, it was not repressed by the caspase family inhibitor Z-DEVD-FMK (Supplementary Figure S2B). ROS regulate Akt phosphorylation in several different cell types (Le Bras et al., 2005; Paravicini and Touyz, 2008; Ramos, 2008). We demonstrated that artocarpin-induced Akt activation was inhibited by preincubation with DPI or MitoTEMPOL (**Figure 4B**). In addition, 10 μM artocarpin significantly decreased U87 cell viability and this effect was abrogated by the Akt inhibitor SH-5 and by LY294002 (**Figure 4C**). We also investigated the role of PI3K/Akt in artocarpin-induced caspase activation and PARP cleavage. **Figure 4D** shows that artocarpin-induced caspase-3, -7, and -9 activation and PARP cleavage were inhibited in U87 cells by pretreatment with LY294002 or SH-5. Therefore, these data suggested that artocarpin induces apoptosis in U87 cells via the ROS/PI3K/Akt signaling pathway.

### ROS Induces ERK1/2 Activation via Akt Resulting in the Apoptosis of U87 Cells

**FIGURE 5:**
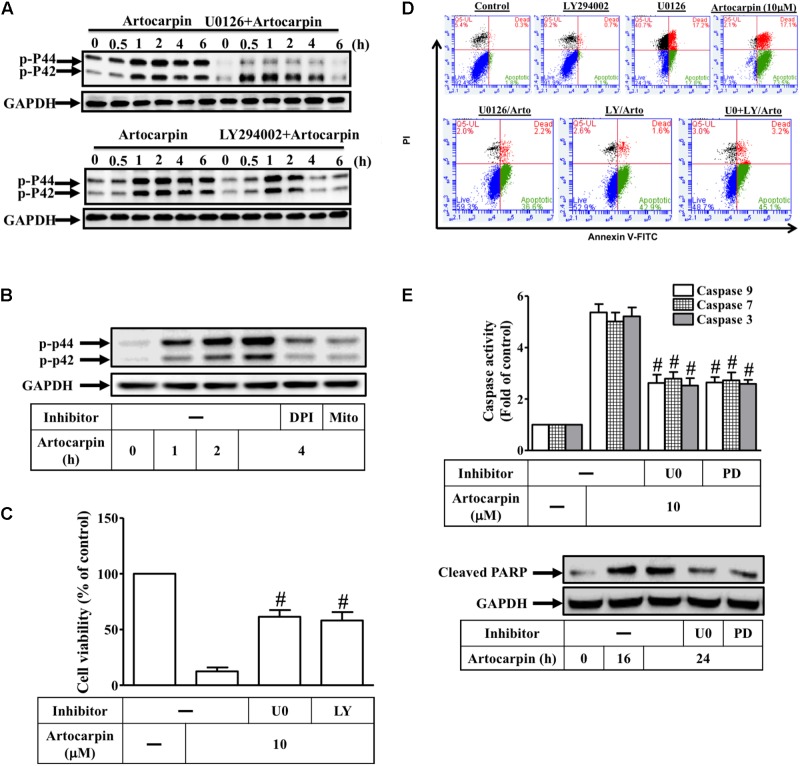
Reactive oxidative species induces ERK1/2 activation via Akt, resulting in the apoptosis of U87 cells. **(A)** Cells were either not pretreated or pretreated with U0126 (10 μM) or LY294002 (10 μM) for 1 h then incubated with artocarpin for the indicated times. Phospho-ERK1/2 protein expression was determined by western blot. **(B)** Cells were either not pretreated or pretreated with DPI (1 μM) or MitoTEMPOL (10 μM) for 1 h then incubated with artocarpin for the indicated times. Phospho-ERK1/2 protein expression was determined by western blot. **(C)** Cells were pretreated with U0126 (10 μM) or PD98059 (10 μM) for 1 h then incubated with artocarpin for 24 h. Cell viability was assayed with MTT. **(D)** Cells were pretreated with U0126 (10 μM) or LY294002 (10 μM) for 1 h then incubated with artocarpin for 24 h. U87 cell apoptosis was evaluated by flow cytometry. U87 cells were labeled with annexin V-FITC and PI. Flow cytometry profile shows annexin-V-FITC staining in the *x*-axis and PI in the *y*-axis. **(E)** Cells were pretreated with U0126 (10 μM) or PD98059 (10 μM) for 1 h then incubated with artocarpin for 24 h. Caspase activity was analyzed with caspase-3, -7, and -9 colorimetric assay kits. Cells were pretreated with U0126 (10 μM) or PD98059 (10 μM) for 1 h then incubated with artocarpin for the indicated times. Cleaved PARP protein expression was determined by western blot. Data are expressed as means ± SE of three independent experiments. ^#^*P* < 0.01 compared with cells exposed to artocarpin alone.

The generation of intracellular ROS significantly affects the involvement of the MAPK pathway in apoptosis signaling (Le Bras et al., 2005; Ramos, 2008; Lee et al., 2011). In this study, ERK1/2 phosphorylation significantly increased 30 min after artocarpin exposure and was sustained for 6 h thereafter (**Figure 5A**). However, Z-DEVD-FMK did not have this effect (Supplementary Figure S2B). Neither JNK1/2 nor p38 MAPK was activated after artocarpin treatment (Supplementary Figure S3). Pretreatment with the MEK1/2 inhibitor U0126 or with LY294002 reduced artocarpin-induced ERK1/2 (**Figure 5A**). U0126 only partially reduces ERK phosphorylation. ROS regulate ERK1/2 phosphorylation in various cell types (Le Bras et al., 2005; Paravicini and Touyz, 2008; Ramos, 2008). Here, we showed that preincubation with DPI or MitoTEMPOL inhibited artocarpin-induced ERK1/2 activation (**Figure 5B**). On the other hand, 10 μM artocarpin significantly decreased U87 cell viability but this effect was abrogated by U0126 or LY294002 (**Figure 5C**). As shown in Supplementary Figure S4C, treatment with inhibitor alone has no significant effects on each experiment. We then investigated whether Akt- and ERK1/2 activation were important in artocarpin-induced apoptosis. Cells were either not pretreated or they were pretreated with U0126 or LY294002, exposed to artocarpin for 24 h, and analyzed for apoptosis. **Figure 5D** shows that U0126 and LY294002 significantly inhibited artocarpin-induced apoptosis. LY294002 or U0126 alone had no significant effect on cell apoptosis. On the other hand, the combination of LY294002 and U0126 did not significantly protect cells from apoptosis. We also explored the role of ERK1/2 in artocarpin-induced caspase activation and PARP cleavage. **Figure 5E** shows that in U87 cells, artocarpin-induced caspase-3, -7, and -9 activation and PARP cleavage were reduced by pretreatment with U0126 or PD98059. Therefore, these data suggested that in U87 cells, artocarpin induces apoptosis via the ROS/PI3K/Akt/ERK1/2 signaling pathway.

### Artocarpin Suppressed Brain Cancer Growth in the Mouse Xenograft Model

**FIGURE 6:**
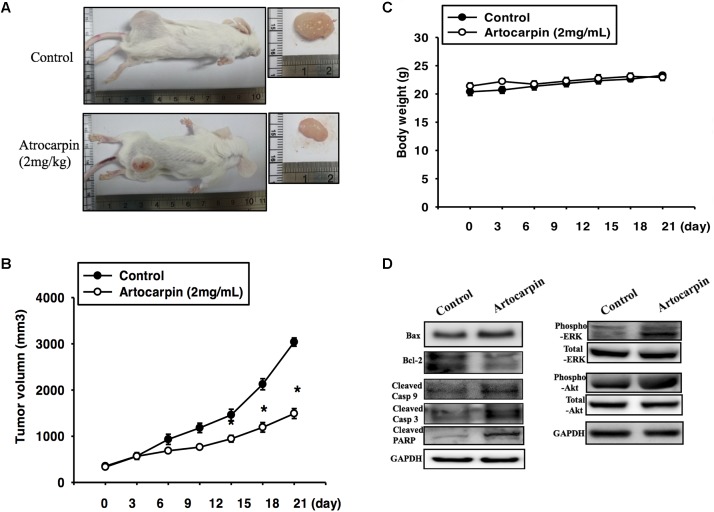
Artocarpin inhibits xenograft tumor growth in SCID mice. **(A,B)** Mice were subcutaneously injected with U87 cells. When the tumors grew to 100 mm^3^, the mice were administered artocarpin (2 mg kg^-1^) or vehicle once daily for 3 weeks. Tumor volume was determined at various times after implantation (*n* = 8–10). **(C)** Mouse body weights were measured at various times after tumor implantation. **(D)** Western blot analyses of Bax, Bcl-2, cleaved caspase-3, cleaved caspase-9, cleaved PARP, phospho-ERK1/2, and phospho-Akt levels in tumors with- and without artocarpin treatment. ^∗^*P* < 0.05.

**FIGURE 7:**
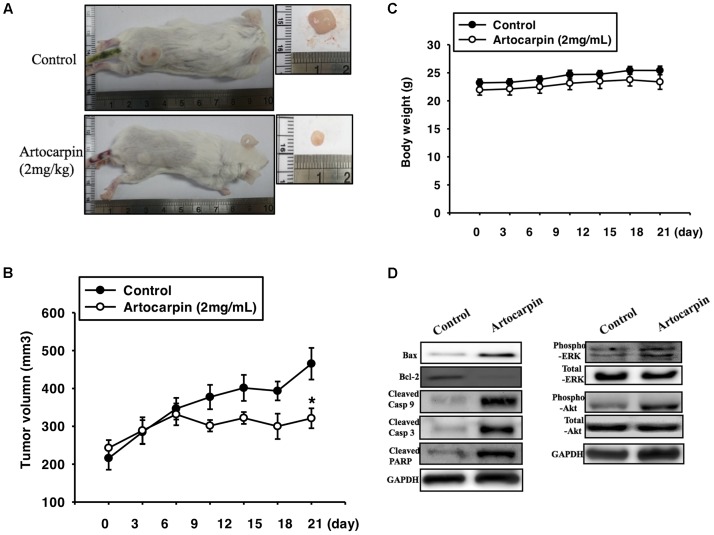
Artocarpin inhibits xenograft tumor growth in SCID mice. **(A,B)** Mice were subcutaneously injected with U118 cells. When the tumors grew to 100 mm^3^, the mice were administered artocarpin (2 mg kg^-1^) or vehicle once daily for 3 weeks. Tumor volume was determined at various times after implantation (*n* = 8–10). **(C)** Mouse body weights were measured at various times after tumor implantation. **(D)** Western blot analyses for Bax, Bcl-2, cleaved caspase-3, cleaved caspase-9, cleaved PARP, phospho-ERK1/2, and phospho-Akt levels in tumors with- and without artocarpin treatment. ^∗^*P* < 0.05.

To determine whether artocarpin had anticancer properties *in vivo*, we implanted xenografts of U87- and U118 cells into SCID mice. We tested both cell lines because they significantly differed in terms of their morphology, histology, and structure. U87 tumors were almost 3× larger than U118 cells (Jaworski et al., 2013). After the xenograft tumors had grown for 14 days, we divided the mice into two groups. One received vehicle and the other was administered artocarpin (2 mg kg^-1^ d^-1^). As shown in **Figures 6**, **7**. Tumor growth was significantly inhibited in the artocarpin-treated group (**Figures 6A**, **7A**). The mean tumor volume in the control group was significantly larger than that of the artocarpin group (**Figures 6B**, **7B**). We excised the tumors and performed western blot analyses on them. Bax, cleaved caspase-3, cleaved caspase-9, cleaved PARP, phospho-ERK1/2, and phospho-Akt expression levels were significantly higher in the artocarpin-treated group than the control group (**Figures 6D**, **7D**). These findings suggest that artocarpin suppresses tumor growth by inducing U87 and U118 cell apoptosis *in vivo*.

## Discussion

In this study, we demonstrated that artocarpin decreased U87- or U118 cell viability by inducing apoptosis. Cell death was associated with caspase activation and PARP cleavage. It was mediated by the mitochondrial pathway. These findings were corroborated by mitochondrial depolarization, cytochrome c release, and downregulation of the antiapoptotic Bcl-2 protein. We also showed that intracellular ROS production activated mitochondrial signaling and resulted in apoptosis after artocarpin treatment. ROS generation led to PI3K/Akt/ERK1/2-induced cell death. These observations were confirmed by *in vivo* studies. These data suggested that artocarpin may generate ROS and induce mitochondrion-associated apoptosis in human glioblastoma cells via pro-oxidative activity.

The biochemical pathways of apoptosis may be extra- or intracellular and caspase-dependent or -independent. In this study, we showed that artocarpin-induced apoptosis in glioblastoma cells is caspase-dependent and is mediated by the activation of the AKT pathway. This finding is in contrast with previous studies which reported that activated AKT signaling compromised the therapeutic effects of the chemotherapeutic agents used in the treatment of GBM (Hirose et al., 2005; Yin et al., 2014). The artocarpin-induced apoptosis observed in the present study proceeded through the intrinsic apoptotic pathway. This observation is compatible with recent preclinical data and suggests that targeting intrinsic apoptosis may be an efficacious approach for the treatment of malignant gliomas (Karpel-Massler et al., 2017). Artocarpin does not affect the viability of normal rat brain cortex astrocytes or mouse microglial cells. Therefore, we have provided supporting evidence for the efficacy of this new therapy and insight into the mechanisms of PI3K/Akt/ERK1/2-induced cancer cell death.

Several studies have indicated that increased ROS production may have anti-cancer effects by inducing apoptosis (Sahu et al., 2009; Lee et al., 2017). ROS can be produced by NADPH oxidases, lipoxygenases, cyclooxygenases, and mitochondria. In this study, we observed that artocarpin-induced ROS production is attenuated by NADPH oxidase inhibitors, mitochondria-targeted antioxidants, and free radical scavengers. Therefore, artocarpin-induced mitochondrial ROS production was driven by NADPH oxidase pathway activation in U87- and U118 cells. Our study demonstrated anti-cancer effects of artocarpin similar to those reported in previous studies (Kim et al., 2013; Tsai et al., 2017).

Mitochondria participate in various physiological and biochemical processes like the tricarboxylic acid cycle, oxidative stress response, and carbohydrate- and fatty acid metabolism (James et al., 2005; Chen Z.P. et al., 2016). Previous studies seldom demonstrated that natural products with anticancer activity induced mitochondria-mediated apoptosis. In GBM cells treated with artocarpin, mitochondrial-mediated apoptosis was regulated by antiapoptotic (Bcl-2, Bcl-Xl, Bcl-1) and proapoptotic (Bax, Bad, and Bak) proteins (Savitskaya and Onishchenko, 2015). Here, we observed that artocarpin induced Bad- and Bax expression and repressed Bcl-2 expression in U87- and U118 cells. Artocarpin also altered mitochondrial membrane permeability, thereby releasing cytochrome c into the cytosol. The effects induced by artocarpin were abrogated by pretreatment with free radical scavengers. Therefore, artocarpin-induced apoptosis proceeds via a ROS-dependent mitochondrial pathway in glioblastoma cells.

Protein kinase B (PKB) is involved in several pathophysiological activities associated with tumor growth and metabolism (Manning and Cantley, 2007). PKB is also known as serine/threonine kinase Akt. It may modulate gene regulation of proteins in apoptotic pathways (Ramos, 2008). Studies have shown that Akt induces cancer cell proliferation and is anti-apoptotic. In our previous study, however, the pretreatment of cells with specific Akt473 inhibitors significantly suppressed the growth of A549 and H1299 cells. Therefore, Akt473 is required for artocarpin-induced apoptosis. In this study, phosphorylation of S473 in Akt occurred during artocarpin-mediated apoptosis. This reaction was reported previously (Hsieh et al., 2010; Tsai et al., 2017; Xie et al., 2017). In the present study, we observed that artocarpin induced Akt activation in glioblastoma cells. This mechanism is regulated by ROS activation and has been demonstrated in earlier studies (Lee et al., 2011; Lee and Yang, 2012, 2013). We also found that artocarpin-induced apoptosis, caspase-3, -7, and -9 activation, and PARP cleavage were reduced by pretreatment with inhibitors of PI3K or Akt. Therefore, artocarpin-induced apoptosis of U87 and U118 cells is mediated by activating the ROS/PI3K/Akt signaling pathway. Akt is a critical regulator of cancer cell proliferation and survival (Song et al., 2005). Nevertheless, to the best of our knowledge, this study is the first to demonstrate that artocarpin-induced apoptosis in glioblastoma cells proceeds by the activation of the Akt pathway. This finding was supported by the fact that the artocarpin-free medium we tested did not activate Akt (Supplementary Figure S2B). In the future, we will attempt to determine the molecular mechanism of artocarpin-induced, Akt-dependent apoptosis in glioblastoma cells.

The generic MAPK signaling pathway can be activated by increases in the levels of intracellular ROS. MAPK interacts with the ERK1/2, JNK1/2, p38 MAPK, and ERK5 cascades (Sun et al., 2015). These may affect apoptosis in various cells (Song et al., 2005). We found that artocarpin induced the activation of ERK1/2 but not JNK1/2 or p38 MAPK in glioblastoma cells. Conversely, we demonstrated that artocarpin-mediated ERK1/2 activation was inhibited by pretreatment with PI3K- and NADPH oxidase inhibitors and by a mitochondria-targeted antioxidant. These results corroborate previous studies which reported that ROS and PI3K/Akt regulate ERK1/2 phosphorylation in various cell types (Lee et al., 2011; Lee and Yang, 2012, 2013). We showed that artocarpin-induced apoptosis, caspase-3, -7, and -9 activation, and PARP cleavage were all repressed by pretreatment with a MEK1/2 inhibitor. Therefore, artocarpin induces apoptosis via the ROS/PI3K/Akt/ERK1/2 signaling pathway in U87- and U118 cells.

## Conclusion

**FIGURE 8:**
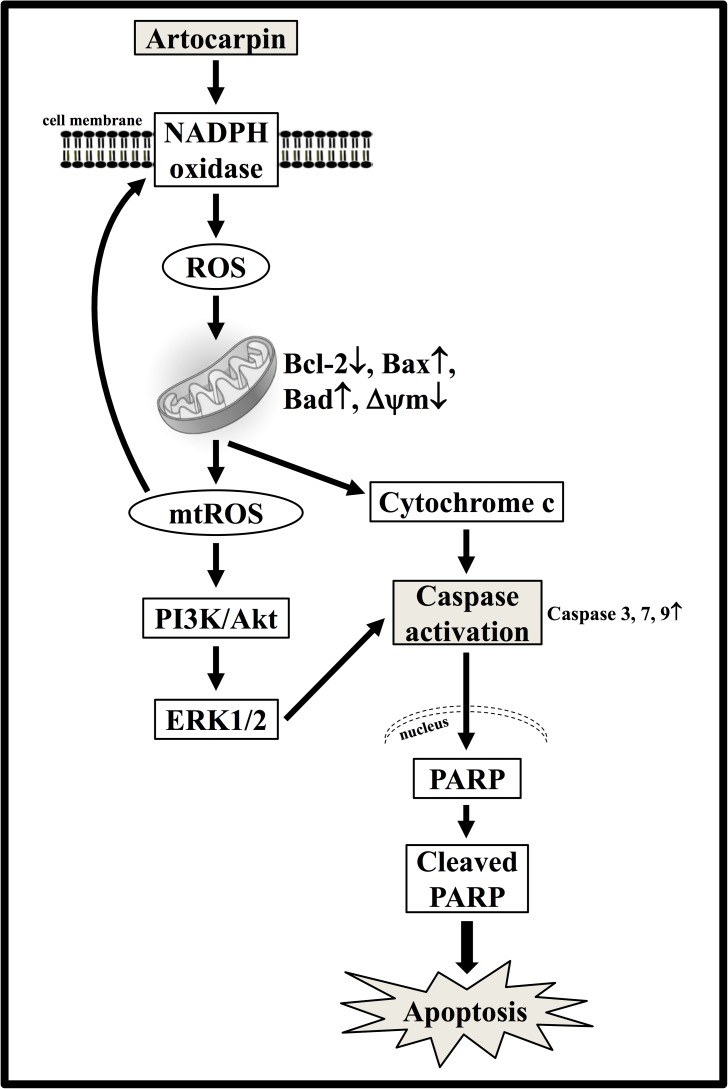
Proposed model for the anticancer activity of artocarpin in U87 cells. Artocarpin decreased U87 cell viability by inducing apoptosis in a time- and concentration-dependent manner. Artocarpin-induced apoptosis was associated with caspase activation and PARP cleavage. It was mediated by the mitochondrial pathway as indicated by mitochondrial depolarization, cytochrome c release, and downregulation of the antiapoptotic Bcl-2 protein. Artocarpin induced intracellular ROS generation. Intracellular ROS production was apparently essential for mitochondrial pathway activation and apoptosis induction following artocarpin exposure. Oxidative stress caused by artocarpin treatment was associated with Akt- and ERK1/2 activation as indicated by Akt- and ERK1/2 phosphorylation. The ROS generated by artocarpin treatment induced cell death by PI3K/Akt/ERK1/2. These findings suggest that artocarpin is a potential chemotherapeutic agent for GBM treatment.

In the present study, we gathered and presented evidence that artocarpin-induced apoptosis in U87- and U118 cells is associated with caspase activation and PARP cleavage and is mediated by the mitochondrial pathway, as indicated by mitochondrial depolarization, cytochrome c release, and downregulation of the antiapoptotic Bcl-2 protein (**Figure 8**). Intracellular ROS production may play a critical role in the activation of the mitochondrial pathway and induction of apoptosis after artocarpin treatment. The oxidative stress induced by artocarpin treatment is associated with Akt- and ERK1/2 activation. Following artocarpin exposure, ROS production results in PI3K/Akt/ERK1/2-induced cell death. Therefore, artocarpin may be an efficacious chemotherapeutic agent for the treatment of GBM.

## Author Contributions

M-HT designed the research, wrote and critically reviewed this manuscript, and finalized this study. C-WL and L-FH performed this study and wrote the initial manuscript. J-FL performed the animal study. I-TL performed the statistical analysis. Y-CC helped the molecular analysis and PCR. M-HL helped in the molecular studies and experiments.

## Conflict of Interest Statement

The authors declare that the research was conducted in the absence of any commercial or financial relationships that could be construed as a potential conflict of interest.

## Abbreviations

APO: apocynin
DPI: diphenyleneiodonium chloride
GBM: glioblastoma multiforme
NAC: *N*-acetyl-L-cysteine
PARP: poly (ADP-ribose) polymerase
PKB: protein kinase B
RNS: reactive nitrogen species
ROS: reactive oxidative species
SRB: sulforhodamine B.
