# Supplementary material for: Extracts of Artocarpus communis Induce Mitochondria-Associated Apoptosis via Pro-oxidative Activity in Human Glioblastoma Cells
Source: Front Pharmacol. 2018 May 2;9:411. doi: 10.3389/fphar.2018.00411 (PMC5941989; doi:10.3389/fphar.2018.00411)
Supplement: Supplementary file 1 [file Data_Sheet_1.doc]

Supplementary Material

**Extracts of *Artocarpus communis* induce mitochondria-associated apoptosis via pro-oxidative activity in human glioblastoma cells**

Chiang-Wen Lee^1,2,3#^; Lee-Fen Hsu^9#^; Ming-Hsueh Lee^6^; I-Ta Lee^4,5^; Ju-Fang Liu^7^; Yao-Chang Chiang^1,8^; Ming-Horng Tsai^10,11*^

^1^Department of Nursing, Division of Basic Medical Sciences, Chang Gung University of Science and Technology, Chia-Yi, Taiwan

^2^Chronic Diseases and Health Promotion Research Center, Chang Gung University of Science and Technology, Chia-Yi, Taiwan

^3^Research Center for Industry of Human Ecology, Chang Gung University of Science and Technology, Taoyuan, Taiwan

^4^School of Medicine, College of Medicine, China Medical University, Taichung, Taiwan

^5^Graduate Institute of Biomedical Sciences, College of Medicine, China Medical University, Taichung, Taiwan

^6^Division of Neurosurgery, Department of Surgery, Chang Gung Memorial Hospital, Chiayi 61363, Taiwan

^7^Central Laboratory, Shin-Kong Wu Ho-Su Memorial Hospital, Taipei, Taiwan

^8^Center for Drug Abuse and Addiction, China Medical University Hospital, China Medical University, Taichung 40447, Taiwan

^9^Department of Respiratory Care, Chang Gung University of Science and Technology, Chiayi Campus, Chiayi, Taiwan

^10^Department of Pediatrics, Division of Neonatology and Pediatric Hematology/Oncology, Chang Gung Memorial Hospital, Yunlin, Taiwan

^11^College of Medicine, Chang Gung University, Taoyuan, Taiwan

#Dr. Lee and Dr. Hsu contributed equally to this study

*Correspondence: Dr. Ming-Horng Tsai.

Department of Pediatrics, Division of Neonatology and Pediatric Hematology/Oncology, Chang Gung Memorial Hospital, Yunlin, Taiwan

No.707, Gongye Rd., Sansheng, Mailiao Township, Yunlin, 638, Taiwan, R.O.C.

E-mail: [mingmin.tw@yahoo.com.tw](mailto:mingmin.tw@yahoo.com.tw), Tel: +886-5-6915151-2878, Fax: +886-5-6913222

**Supplementary Materials & Methods**

**Reagents**

MitoTempo (a specific scavenger for mitochondrial superoxide anions) was obtained from Cayman (Ann Arbor, MI, USA). MitoSOX Red mitochondrial superoxide indicator was purchased from Molecular Probes, Eugene, OR, USA. Caspase-3, -7, and -9 colorimetric assay kits were obtained from R&D Systems Inc. (Minneapolis, MN, USA). PE anti-human CD253 (TRAIL) was from Biolegend (#308206)( San Diego, CA, USA).

***Measurement of mitochondrial-derived ROS production***

We washed U87 cells with warm HBSS and incubated them in cell medium containing 5 μM MitoSOX Red mitochondrial superoxide indicator at 37°C for 30 min. Then, HBSS or medium containing MitoSOX Red mitochondrial superoxide indicator was removed and replaced with fresh medium. U87 cells were pre-incubated with inhibitors and then treatment with artocarpin for 24hr. We washed the cells twice with PBS and detached them with EDTA/trypsin, and used a FACScan flow cytometer (BD Biosciences, San Diego, California, USA) to analyze the fluorescence intensity of the cells at 510 nm excitations and 580 nm emissions.

***Caspase activity determinations***

We used the caspase-3, -7, and -9 colorimetric assay kits and manufacturer’s protocols (R&D Systems Inc., MN, USA) to measure the caspase activity from cell lysates. U87 Cells were treated with various inhibitors including SH-5, LY294002, PD98059 and U0126 for 24 h, and then we used lysis buffer to treat the cells [50 mM Tris-HCl (pH 7.4), 2 mM DTT, 1 mM EDTA, 10 mM digitonin, and 10 mM EGTA]. We used Ac-DEVD-pNA and Ac-LEHD-pNA as substrates of casepase-3, -7, and -9 to incubate the cell lysates at 37°C for 1 h. We measured caspase activity and absorbance with an enzyme-linked immunosorbent assay reader at OD_405_. We performed three independent experiments to obtain the results and analyses.

***Cell viability***

We measured cell viability according to the formation of formazan, a blue product resulting from the metabolism of a colorless substrate by mitochondrial dehydrogenases. U87 cells (2.5×10^5^ per well in a 24-well plate) were incubated at 37°C with various inhibitors including SH-5, LY294002, PD98059 and U0126 for 24 h, and we treated these cells with an MTT solution (5 mg/ml) for 2 h. We used a microplate reader to measure the dark blue formazan crystals, which were formed in intact cells dissolved in DMSO and the resultant absorbances at 540 nm. We expressed the results as the percentage of metabolized MTT relative to that of control cells which were determined by absorbance measurements.

***Measurement of intracellular ROS accumulation***

CellROX Green Reagent (Molecular Probes, Eugene, OR) is a novel fluorogenic probe for measuring oxidative stress in live cells. We detected the fluorescence for CellROX Green Reagent staining at 485/520 nm, and we used a fluorescence microscope (Zeiess, 200M, Axiovert, Germany). In order to obtain the results, we washed U87 cells with warm PBS and incubated these cells in HBSS which contained 5 μM CellROX Green Reagent in 30°C for 30 min. Then, HBSS or medium with CellROX Green Reagent was taken out and we then used fresh medium. We incubated U87 cells with various inhibitors including MCI-186, N-acetyl-L-cysteine (NAC), MitoTEMPOL and diphenyleneiodonium chloride for 6 h. We used PBS to wash cells twice and detached these cells with trypsin/EDTA, and then used a FACScan flow cytometer (BD Biosciences, San Diego, California, USA) to detect the fluorescence intensity of the cells at 485 nm excitations and 520 nm emissions.

*Preparation of cell extracts and Western blot*

We grew the U87 cells to confluence in a 6-well plate, and treated these cells with artocarpin (10 μM) for a series of different time intervals. The collected cells were lysed with ice-cold lysis buffer containing 25 mM Tris-HCl (pH 7.4), 25 mM NaCl, 25 mMNaF, 25 mM sodium pyrophosphate, 1 mM sodium vanadate, 2.5 mM EDTA, 0.05% (w/v) Triton X-100, 0.5% (w/v) sodium dodecyl sulfate (SDS), 0.5% (w/v) deoxycholate, 0.5% (w/v) NP-40, 5μ g/ml leupeptin, 5μ g/ml aprotinin, and 1 phenylmethylsulfonyl fluoride (PMF). Lysates were centrifuged at 45,000× g for 1 h at 4 °C to yield the whole cell extract similar protocol previous studies [21]. Samples were denatured, subjected to SDS-PAGE using a 12% running gel, and transferred to nitrocellulose membrane. We incubated the membranes with anti-phosphorylation Akt, anti-phosphorylation P44/P42, anti-phosphorylation PJNK1/PJNK2, anti-phosphorylation P38 or anti-cytochrome C antibody for 24 h, and incubated the membranes with anti-mouse or anti-rabbit horseradish peroxidase antibody for 1 h. We used ECL reagents were from PerkinElmer (Waltham, MA, U.S) to detect the immunoreactive bands, which were developed by Hyperfilm-ECL was from PerkinElmer (Waltham, MA, U.S).

***Flow cytometry analysis of TRAIL expression***

Flow cytometric assessment of cell death was performed using the PE anti-human CD253 (TRAIL) to quantify cell death receptor. Briefly, the growing U87 cells were digested with 0.25% trypsin first and counted. We diluted these cells to a final concentration of 1×10^5^ cells/ml and inoculated them in a culture dish at 10 ml/dish and treated with artocarpin for the indicated times. The cell pellet was collected and re-suspended in 100mL Phosphate-Buffered Saline (PBS). PE anti-human CD253 (TRAIL) was then added to the cell suspension. After incubation at room temperature for 30 minutes, the stained cells were analyzed by flow cytometry (FACScan flow cytometer, BD, Franklin Lakes, NJ, USA) at a wavelength of 488nM.

# Supplementary Figures and Tables


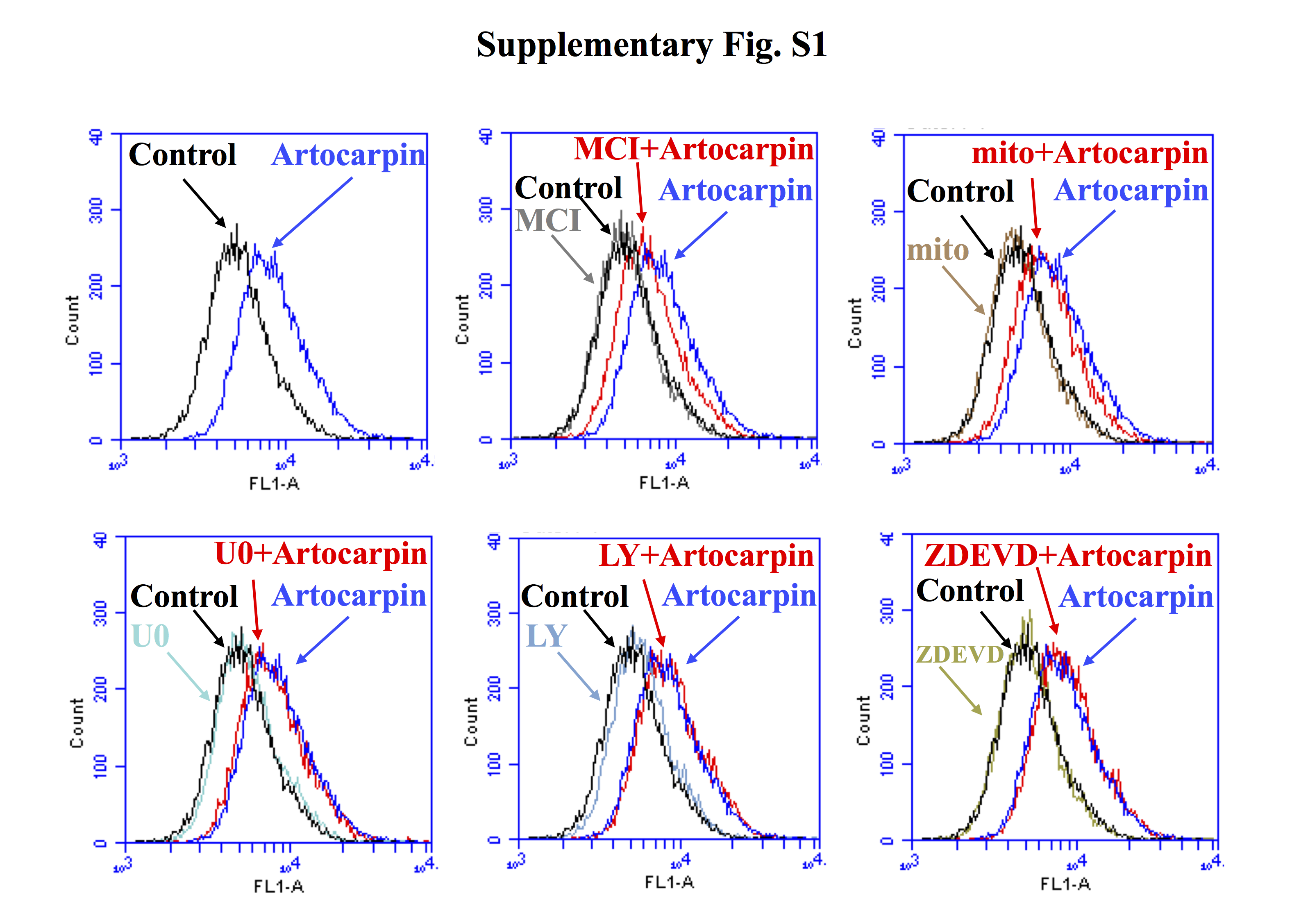


**Supplementary Fig. 1. Effect of mitoTEMPO on artocarpin-induced long-term production of mitochondrial ROS in U87 cells. Confluent cells were labeled with MitoSox (5nM) and then pre-incubated with or without mitoTEMPO (1μM), MCI-186 U0126, LY294002 and zVAD. After incubation for 1h, cells were stimulated with artocarpin (10μM) for 24h. The mitochondrial ROS production was measured using flow cytometry.**


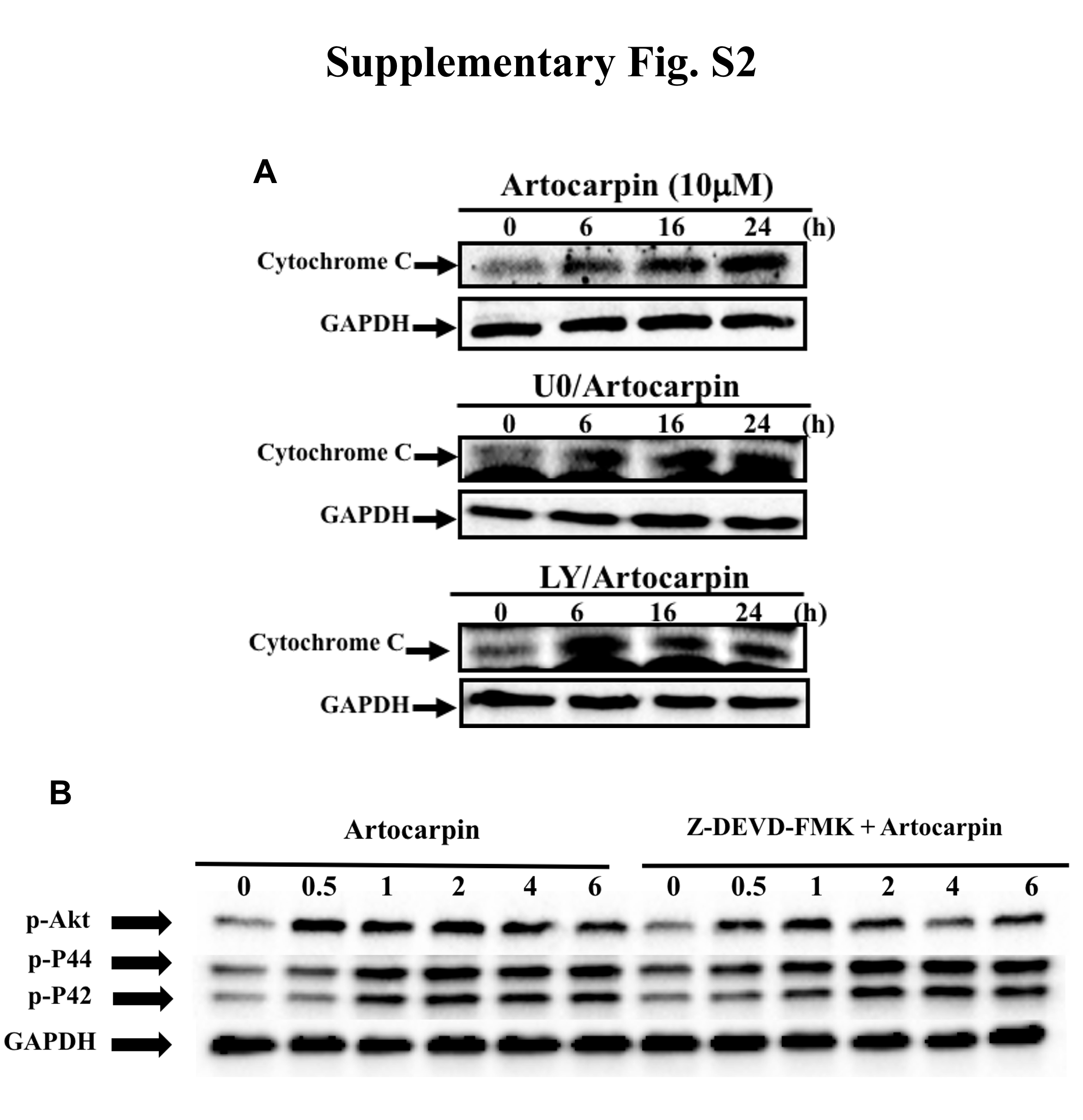


**Supplementary Fig. 2. (A)** Cells were pretreated without or with U0126 (10 μM) or LY294002 (10 μM) for 1 h, and then incubated with artocarpin for the indicated times. The protein expression of Cyotchrome C was determined by Western blot. **(B)** Cells were pretreated with Z-DEVD-FMK (25μM) for 1 h, and then incubated with artocarpin for the indicated times. The protein phosphorylation of Akt or ERK1/2 was determined by Western blot.


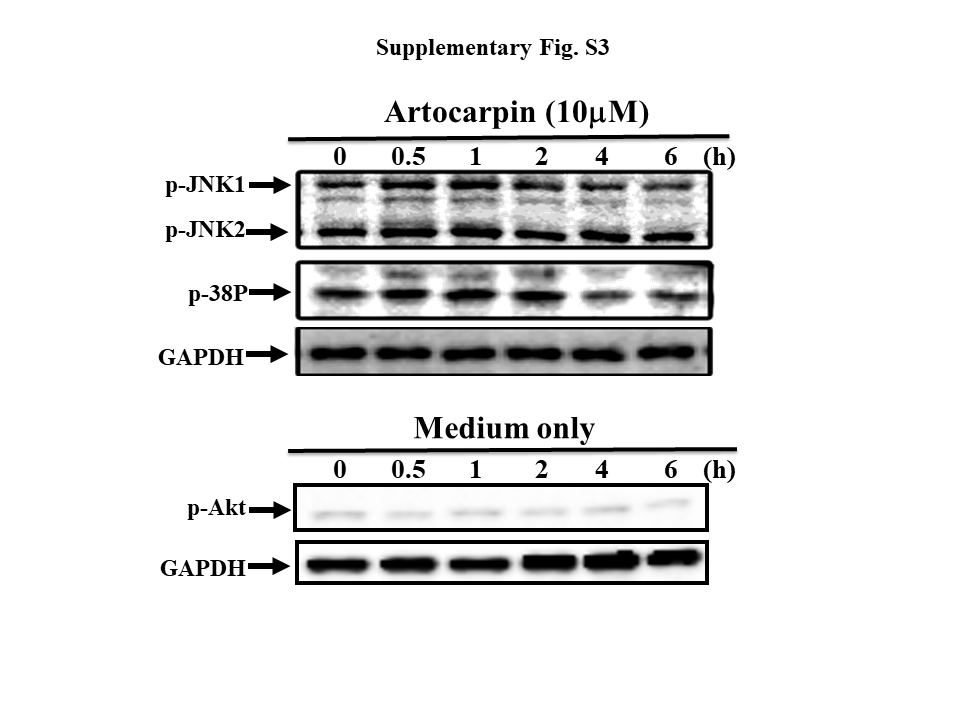


**Supplementary Fig. 3.** Cells were treated with artocarpin or medium for the indicated times. The protein phosphorylation of JNK1/2, P38 and Akt was determined by Western blot.


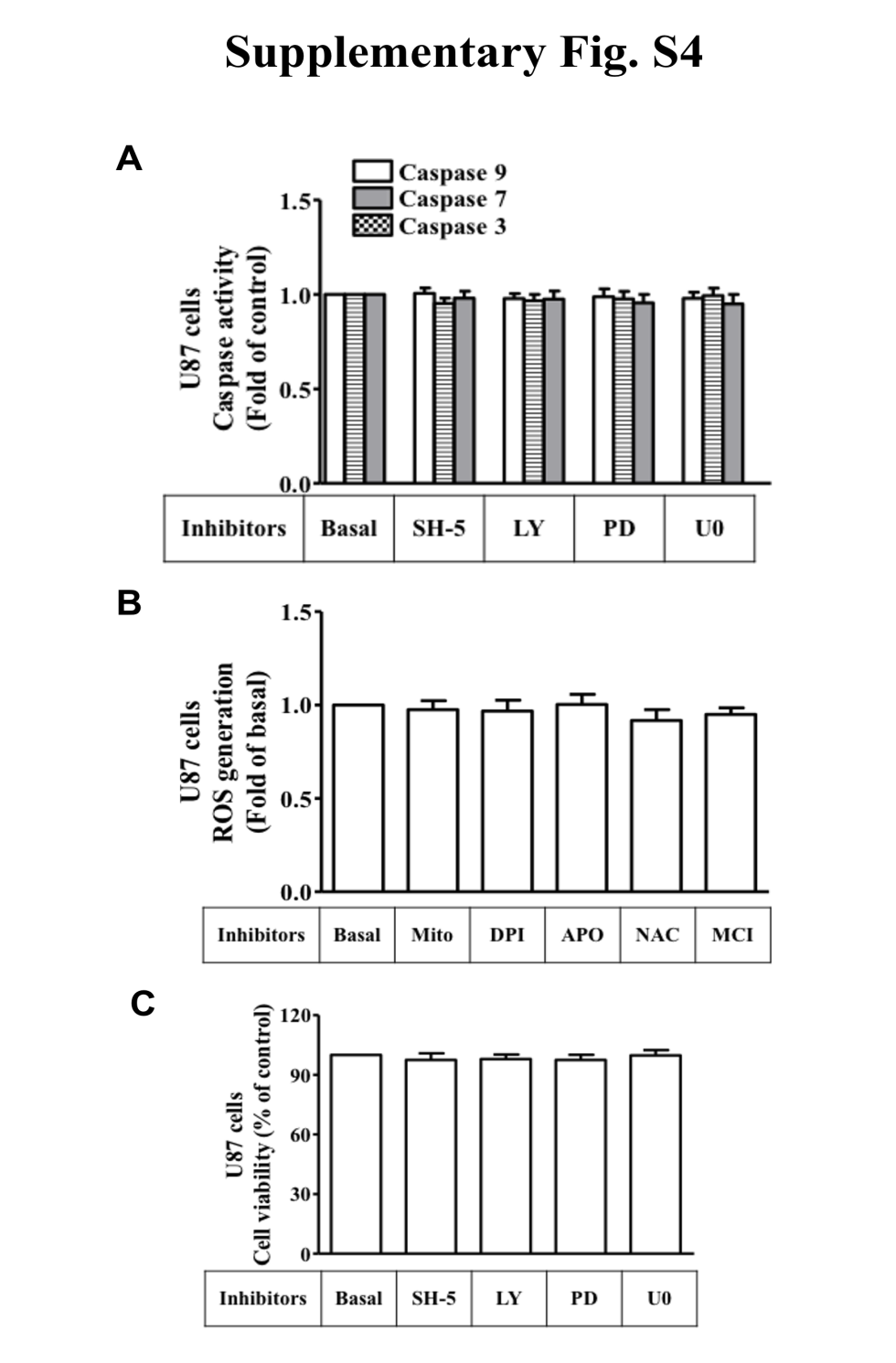


**Supplementary Fig. 4. (A)** Cells were pretreated with SH-5, LY294002, PD98059 and U0126 for 24h. The caspase activity was analyzed by using caspase-3, -7, and -9 colorimetric assay kits. **(B)** Cells were treated with MitoTEMPOL (10 μM), DPI (1 μM), APO (100 μM), NAC (5 mM), or MCI-186 (10 μM) for 24h. The ROS generation was measured using the CellROX reagent. **(C)** Cells were treated with Cells were pretreated with SH-5, LY294002, PD98059 and U0126 for 24h. Cell viability was assayed using the MTT assay.


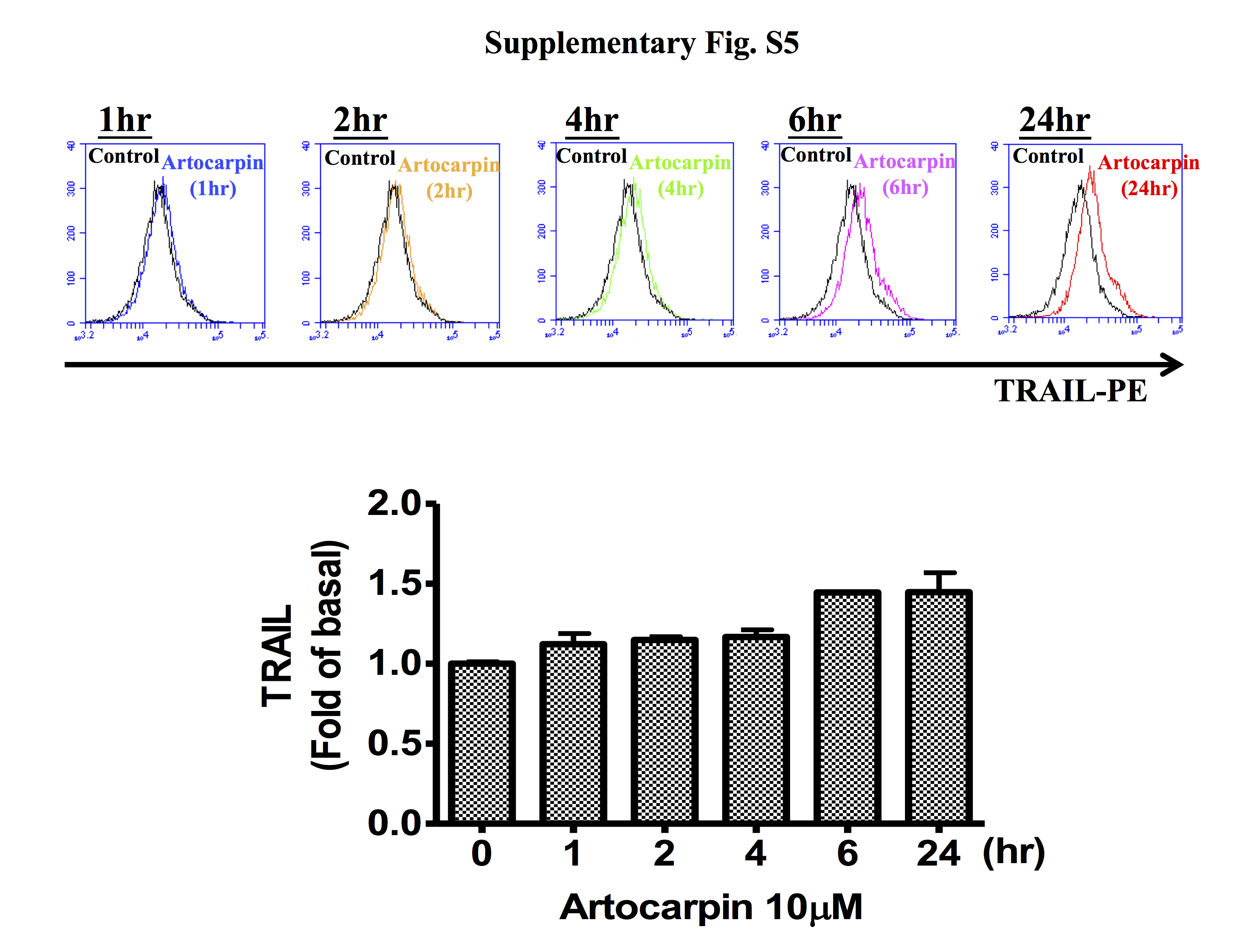


**Supplementary Fig. 5. Artocarpin-induced TRAIL expression in U87 cells. Confluent cells were labeled with PE anti-human CD253 (TRAIL) and thencells were stimulated with artocarpin (10μM) for various times. The TRAIL expression was measured using flow cytometry.**
